# Evidence for plant-derived xenomiRs based on a large-scale analysis of public small RNA sequencing data from human samples

**DOI:** 10.1371/journal.pone.0187519

**Published:** 2018-06-27

**Authors:** Qi Zhao, Yuanning Liu, Ning Zhang, Menghan Hu, Hao Zhang, Trupti Joshi, Dong Xu

**Affiliations:** 1 Department of Computer Science and Technology, Jilin University, Changchun, Jilin, China; 2 Department of Electrical Engineering and Computer Science, and Christopher S Bond Life Sciences Center, University of Missouri, Columbia, Missouri, United States of America; 3 Sino-Dutch Biomedical and Information Engineering School, Northeastern University, Shenyang, Liaoning, China; 4 MU Informatics Institute, University of Missouri, Columbia, Missouri, United States of America; 5 Department of Biostatistics, Brown University, Providence, Rhode Island, United States of America; 6 Department of Molecular Microbiology and Immunology, School of Medicine, University of Missouri, Columbia, Missouri, United States of America; The University of Oklahoma Health Scieces Center, UNITED STATES

## Abstract

In recent years, an increasing number of studies have reported the presence of plant miRNAs in human samples, which resulted in a hypothesis asserting the existence of plant-derived exogenous microRNA (xenomiR). However, this hypothesis is not widely accepted in the scientific community due to possible sample contamination and the small sample size with lack of rigorous statistical analysis. This study provides a systematic statistical test that can validate (or invalidate) the plant-derived xenomiR hypothesis by analyzing 388 small RNA sequencing data from human samples in 11 types of body fluids/tissues. A total of 166 types of plant miRNAs were found in at least one human sample, of which 14 plant miRNAs represented more than 80% of the total plant miRNAs abundance in human samples. Plant miRNA profiles were characterized to be tissue-specific in different human samples. Meanwhile, the plant miRNAs identified from microbiome have an insignificant abundance compared to those from humans, while plant miRNA profiles in human samples were significantly different from those in plants, suggesting that sample contamination is an unlikely reason for all the plant miRNAs detected in human samples. This study also provides a set of testable synthetic miRNAs with isotopes that can be detected *in situ* after being fed to animals.

## Introduction

Since the first microRNA (miRNA) was discovered in *C*. *elegant* [1, 2], more and more miRNAs have been identified in both plants and animals. MiRNA is a type of small non-coding RNA derived from the primary miRNA hairpin of transcripts, with a typical length of 22 nucleotides (nt) in animals and 21 nt in plants. They play important post-regulation roles in a wide range of fundamental biological processes [3] and a variety of diseases [4]. miRNAs function by binding to complementary sequences in the 3' untranslated region (UTR) of protein-coding genes and inducing mRNA cleavage or translational repression [5]. Biogenesis and the mechanism of miRNA action have a high similarity between animals and plants at the molecular level, along with slight differences due to the evolutionary divergence [3]. miRNA is still a hot topic of research, and many new hypotheses and tools are being proposed [6–10].

Recently, an xenomiR hypothesis was proposed wherein plant-derived miRNAs could survive the animal digestion system, be absorbed and transferred into the blood, circulate through an animal’s body, regulate animal gene expression as endogenous miRNAs and induce different phenotypes. This hypothesis was first suggested by Zhang [11], which showed plant-derived miRNAs could exist in humans and mice, and these miRNAs could regulate gene expression in mice, but the results could not be reproduced in several studies [12–15]. Subsequently, many groups tried to detect plant miRNAs in animals after feeding them different diets. Some of them identified plant miRNAs in human or animal body fluids/tissues [16–32], while others failed [33–35]. Some other studies also found that plant-derived miRNAs (such as HJT-sRNA-m7, miR59, and honeysuckle-MIR9211) might prevent or treat human diseases, such as pulmonary fibrosis, breast cancer or influenza [17, 22, 25, 26, 30]. As discussed in [36–56], the key controversy is on whether the plant-derived miRNAs detected in animal samples were contamination during experiments or *bona fide* xenomiRs. Zhang et al. [12] reported that, unlike the results of [11], they detected few miR168, but many other plant-derived miRNAs in insects. And their results also showed that some of the plant miRNAs detected in insects could not be detected in their diet and some plant miRNAs were also detected in non-feeding neonate insects. They indicated that the plant miRNAs detected in their experiments were from the cross-contamination caused by other samples. Snow et al. [13] reported that plant-derived miRNAs (miR156, miR159, and miR169) were indistinguishable from the background signal in qRT-PCR experiments, which were conducted with samples from human, mouse and bees. Witwer et al. [14] reported that the level of plant-derived miR156, miR166, miR167, and miR168 in two primate plasma samples had no indication of response to diet-feeding time, and the low level of plant miRNAs that they detected could be the result from a non-specific amplification. Dickinson et al. [15] reported that the plant miRNAs in mice plasma or liver after ingesting a plant-enriched diet were indistinguishable from the background signal, and the detected plant miRNA might be explained by sequencing errors or cross-contamination.

Two more recent studies used public databases to investigate plant-derived xenomiRs. Kang et al. [57] first comprehensively studied the exogenous miRNA in 824 public human sequencing data sets, and they claimed xenomiRs were from technical artifacts. In their study, the sequencing reads were determined to be clade-specific after adapter removal, read quality control and length control. However, the plant miRNAs belonging to multiple clades were found in single sequencing data, which may not be well explained by contamination. Another recent study by Zheng et al. [58] developed an on-line exogenous miRNA analysis tool, but it is only practical for small-scale analysis due to the large size of sequence data and the slow computing speed. Furthermore, statistical analyses performed in both studies [57, 58] were not rigorous enough.

Although these negative results have demonstrated that contamination of samples or errors in sequencing indeed exist, they could not rule out the possibility of plant-derived xenomiR. To determine whether plant-derived miRNAs truly exist in human bodies, a large-scale analysis of many mammal samples with rigorous statistical analysis is necessary to validate or disprove the plant-derived xenomiR hypothesis. In this study, we analyzed a large number of public small RNA sequencing data obtained from a variety of experiments by different laboratories. We developed an analysis pipeline, and extracted reliable plant miRNA reads from different human body fluid/tissue samples. Subsequently, based on comprehensive plant-derived miRNA profiles, some further bioinformatics and statistical analyses were performed, which yielded reliable evidence to support the plant-derived xenomiR hypothesis. As negative controls, we compared the miRNAs identified in plants and microbiome. Plant-derived miRNAs have a significantly different distribution from the distribution found in plants, and plant-derived miRNAs were rarely found in microbiome. Taken together, we reached the conclusion that plant-derived xenomiR is the most likely explanation based on the analysis of a large number of public small RNA sequencing data. This study provided the first statistically solid evidence using large-scale data to validate the plant-derived xenomiR hypothesis in human.

## Materials and methods

### Data collection

Unlike the study by Kang et al. [57], we collected all human small RNA sequencing data from the National Center for Biotechnology Information Gene Expression Omnibus (NCBI GEO) [59] satisfying three stringent criteria as follows.

1. Small RNA sequencing data are from normal human serum, plasma, plasma exosomes, milk, RBC, platelets, kidney, liver, pancreas, bladder, and thyroid fresh samples.
2. The number of samples from each type of body fluids/tissues should be more than three.
3. Raw sequencing data is required except for red blood cell (RBC) samples.

Raw sequencing data are convenient for controlling read quality, but for RBC samples, this was not required because no plant miRNA was detected even though any quality of reads were accepted. We avoided samples from the skin, digestive tract, respiratory track or immune-related tissues which could contain exogenous RNA sequences inherently. Human samples not for disease studies were collected because diseased tissues may contain mutations, especially in tumor tissues, which may affect the results of plant-derived miRNA screening. And some plant-derived miRNAs in human tissues were also shown to be disease specific [22]. Sample names and their references are listed in S1 Table, and the corresponding data are convenient to download from GEO.

Plant and human miRNA data were downloaded from the miRBase [60]; Human rRNA data was downloaded from Silva rRNA database [61]; All the other human ncRNA data was downloaded from Ensembl [62]; Human mRNA data was downloaded from GENCODE [63]. All the human RNA data were combined with the corresponding RNA information in the latest version of human genome (GRCh38.p5, containing mitochondrial genome) [64]. The human-related microbiome genomes were downloaded from HMPGD [65] including eukaryotes, archaea, bacteria and viruses. MiRNA expression profile data of *Arabidopsis lyrata* (aly), *Arabidopsis thaliana* (ath), *Nicotiana tabacum* (nta), *Oryza sativa* (osa), *Triticum aestivum* (tae) and *Zea mays* (zma) were downloaded from the miRBase. Yeast and *E*. *coli* small RNA sequencing data were downloaded from NCBI GEO [59] and their file names and references are listed in S2 Table. The genome data of yeast and *E*. *coli* were downloaded from NCBI. The RNA data of yeast and *E*. *coli* were extracted from their genomes. The ath small RNA sequencing data were also downloaded from NCBI GEO, and their file names and references are listed in S3 Table. All the data analyzed in the paper can be found at the website http://digbio.missouri.edu/Qi/miRNA/index.html, and a shell script was also provided at the same website for automatically downloading all small RNA sequencing data used in this study.

The only difference between serum and plasma is that the serum includes fibrinogens while plasma does not. RNA sequencing data only characterizes RNA; hence, small RNA sequencing data of serum and plasma should be the same. Therefore, in this study, serum and plasma were regarded as one type of body fluid unless stated otherwise.

### Processing pipeline

**Fig 1:**
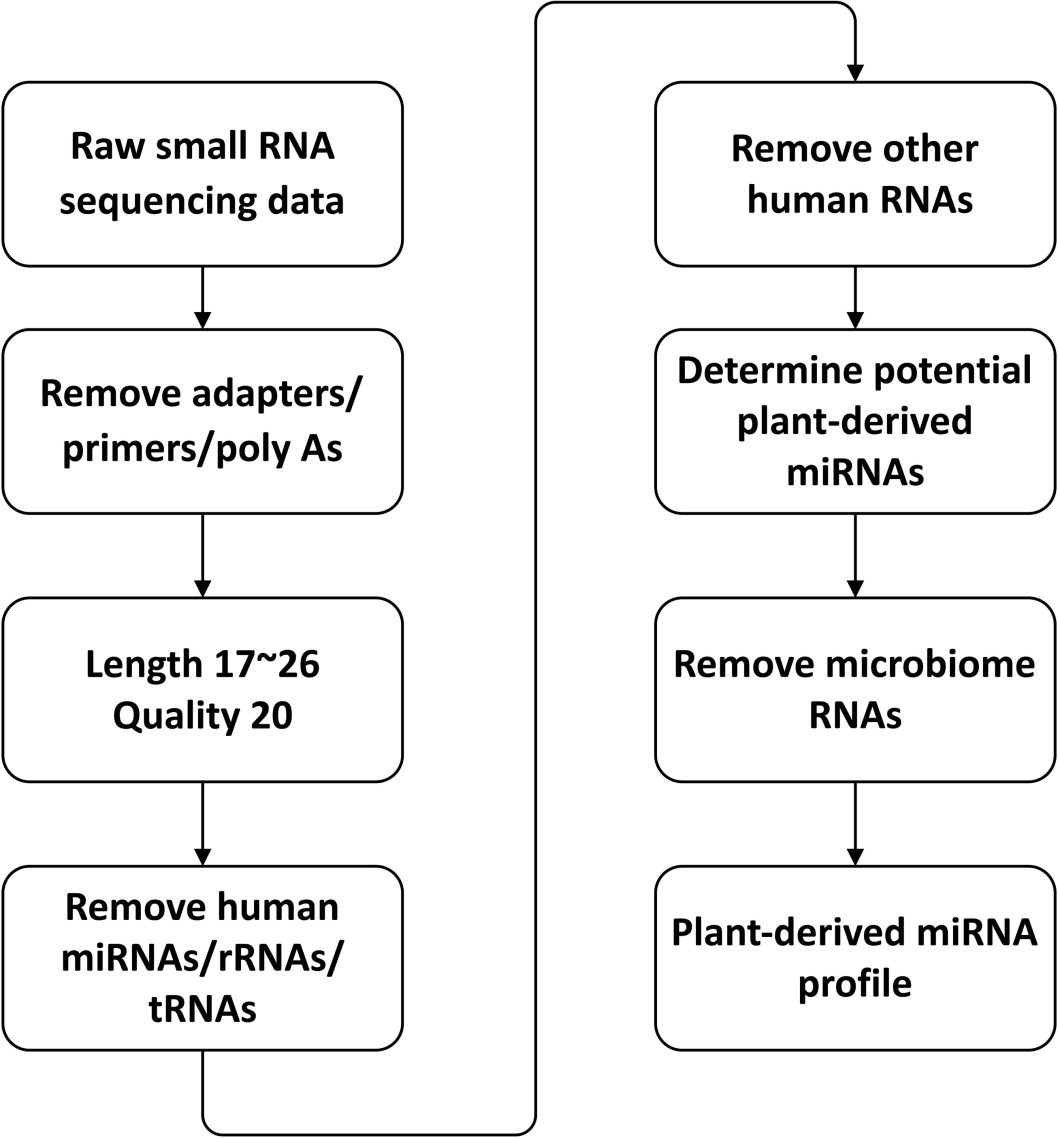
Pipeline for identifying potential plant-derived xenomiR in human samples.

A processing pipeline used for detecting plant miRNAs in human samples is shown in Fig 1. First, the artificial contaminations including adapters, primers and poly A were removed from the sequencing data, and then reads with lengths shorter than 17 nt or longer than 26 nt were removed (17 nt is the shortest plant miRNA and 26 nt is the longest plant miRNA presented in the miRBase). The reads with a quality less than 20 were removed from the data using [66], and the clean reads were obtained for further analyses. The clean reads were aligned to human miRNA/rRNA/tRNA allowing two mismatches and the unaligned reads were aligned to other types of human RNA including all the ncRNA and mRNA allowing one mismatch. The unaligned reads were aligned to plant miRNAs using the following three criteria:

1. Less than or equal to one mismatch.
2. No ‘N’ bases in the reads.
3. The length of a read is equal to that of the aligned plant miRNA.

When processing the RBC samples, “less than two mismatches” was required. The aligned reads were collected and then aligned to human-related microbiome genomes. Finally, the plant miRNA profiles were calculated using the number of unaligned reads. For yeast and *E*. *coli* samples, the processing pipeline is similar to the pipeline above with slight differences which were 1) yeast and *E*. *coli* RNAs were used instead of human RNAs. 2) reads were not aligned to human related microbiome genomes. The abundance of plant miRNA was measured by modified transcripts per million (TPM) *P / T* x 10^6^ where *P* is the number of potential plant miRNA reads determined by our pipeline, and *T* is number of clean reads.

### Abundance distribution fitting of plant miRNAs detected in human and microbiome samples

We assume the denary logarithm of plant miRNA abundances of human, yeast and *E*. *coli* samples follow the Gaussian distribution. The plant miRNA abundance value of each sample was taken as one log10 after adding a very small pseudo-abundance of 10^−3^. Then the abundance data were fitted with Gaussian distributions using the maximum likelihood estimation method.

### Principal component analysis (PCA) and hierarchical clustering

In both PCA and hierarchical clustering, only 24 types of plant miRNA with abundance more than 0.05 (S4 Table) were used for analysis. Abundance values of plant miRNAs both in human and plant samples were normalized by the summation of total plant miRNA abundances to make them comparable, and they were centered and scaled by each type of miRNA. Then, PCA was performed based on these abundance values. We grouped the plant miRNA profiles by the body fluids/tissues, and the average abundance was calculated for each miRNA. After that, the miRNA abundances were scaled according to each type of miRNA. Then, these samples were clustered with the Euclidean distance, using the complete linkage clustering algorithm.

### Marginal two-sample rank sum test

Plant miRNA profiles in plant and human can be regarded as two groups. We standardized the data within groups beforehand to make the values were on the same scale. An observation of an miRNA profile can be regarded as a vector of quantified measurements of plant miRNA which together describe the composition of the sample. Thus, we used multivariate data, i.e., vector valued observations, in the analysis. Since no parametric distribution can be assumed, we used a nonparametric approach, two-sample rank sum test [67], to test the null hypothesis that the distribution of plant miRNA profiles in the plant is the same as that in human.

Let X_1_,X_2_,…,X_*N*_be the combined standardized samples. For each observation, there is a spatial centered rank:

$${\text{R}}_{i}=m_{j}[S(A_{x}({\text{X}}_{i}-{\text{X}}_{j}))],\;i,j=1,2,\ldots ,N,$$

where *m*[*z*] denotes “the mean of *z* over sample *I* = 1,2,…*N*”, *m*_*j*_[*z*]means the same, but the average over index *j*, *S*(*z*) denotes “the sign of *z*” (+ or -) and Ax is chosen to make the ranks satisfy the property

$$\lambda m[R_{i}R_{i}{}^{T}]=m[R_{i}{}^{T}R_{i}]I_{p}.$$

The two-sample spatial rank test statistic is

$$U^{2}=\frac{\lambda }{m[||R_{i}|{|}^{2}]}\sum_{s=1}^{2}n_{s}||{\bar{R}}_{s}|{|}^{2},$$

where ${\bar{R}}_{s}$ is the mean vector of the spatial centered ranks for *s =* 1, 2. The test statistic is asymptotically distribution-free. The p-value of the test statistic is the expected probability that $U_{P}^{2}\geq U^{2}$, where *P* = (*P*_1_,…*P*_N_) is uniformly distributed over the *N*! permutations of (1,…,*N*) and $U_{P}^{2}$ is the value of the test statistic for the permuted samples. *X*_*P*_1__,*X*_*P*_2__,…,*X*_*P*_*N*__

### Pearson correlation coefficients

Pearson correlation coefficients were calculated between two body fluids/tissues based on plant-derived miRNA profiles. To rule out the effects of technical repeats from the same individual, we only calculated the Pearson correlation coefficients between a pair of samples from different laboratories. Then the correlation coefficients were divided into two subsets based on whether the corresponding two samples were from the same body fluids/tissues or not.

## Results

### Identification of plant-derived miRNA in human body fluids/tissues

We selected small miRNA sequencing raw data from healthy humans. A total of 388 public small RNA sequencing data (Tables 1, S5 and S1) of multiple human body fluids/tissues (bladder, brain, breast milk, kidney, liver, pancreas, plasma exosomes, platelet, RBC, serum/plasma, and thyroid) were selected according to our stringent criteria (see “Materials and Methods”). Unless stated otherwise, serum and plasma were regarded as one type of body fluid in our study.

**Table 1:** Human sample list.

Body fluids/Tissues	No. of samples	No. of samples containing plant miRNA	No. of reads mapped to plant miRNA (Average abundance)
Bladder	10	10 (100%^a^)	460 (3.20)
Brain	52	32 (61.5%)	169 (0.49)
Kidney	21	12 (57.1%)	3906 (15.42)
Liver	40	4 (10%)	188 (0.42)
Milk	4	4 (100%)	2759 (27.08)
Pancreas	11	6 (54.5%)	67 (0.87)
Plasma exosomes	147	63 (42.9%)	830 (3.59)
Platelet	6	5 (83.3%)	10 (0.33)
Red blood cell	16	0 (0%)	0 (0)
Serum/Plasma	70	62 (83.3%)	3828 (23.14)
Thyroid	11	4 (36.4%)	9 (0.24)
**Sum**	**388**	**202 (52.1%)**	**12,226 (6.80)**

^a^ Values in the brackets were the percentage of samples containing plant miRNAs.

To carefully eliminate all the reads possibly originated from human or potential contamination, a stringent pipeline was constructed (see “Materials and Methods”). In this pipeline, after removing the low-quality reads, the reads from artificial contamination and the reads originated from human, the remaining reads were aligned to plant miRNAs with stringent criteria to get potential plant miRNAs. These potential plant miRNAs were aligned to human-related microbiome genomes and reads with 0 or 1 mismatch were removed. Finally, 12,226 reads in our 388 samples were kept, covering 166 types of plant miRNA sequences, and their abundances in each sample were obtained as shown in Table 1.

### Distribution of plant-derived miRNA in human samples

**Fig 2:**
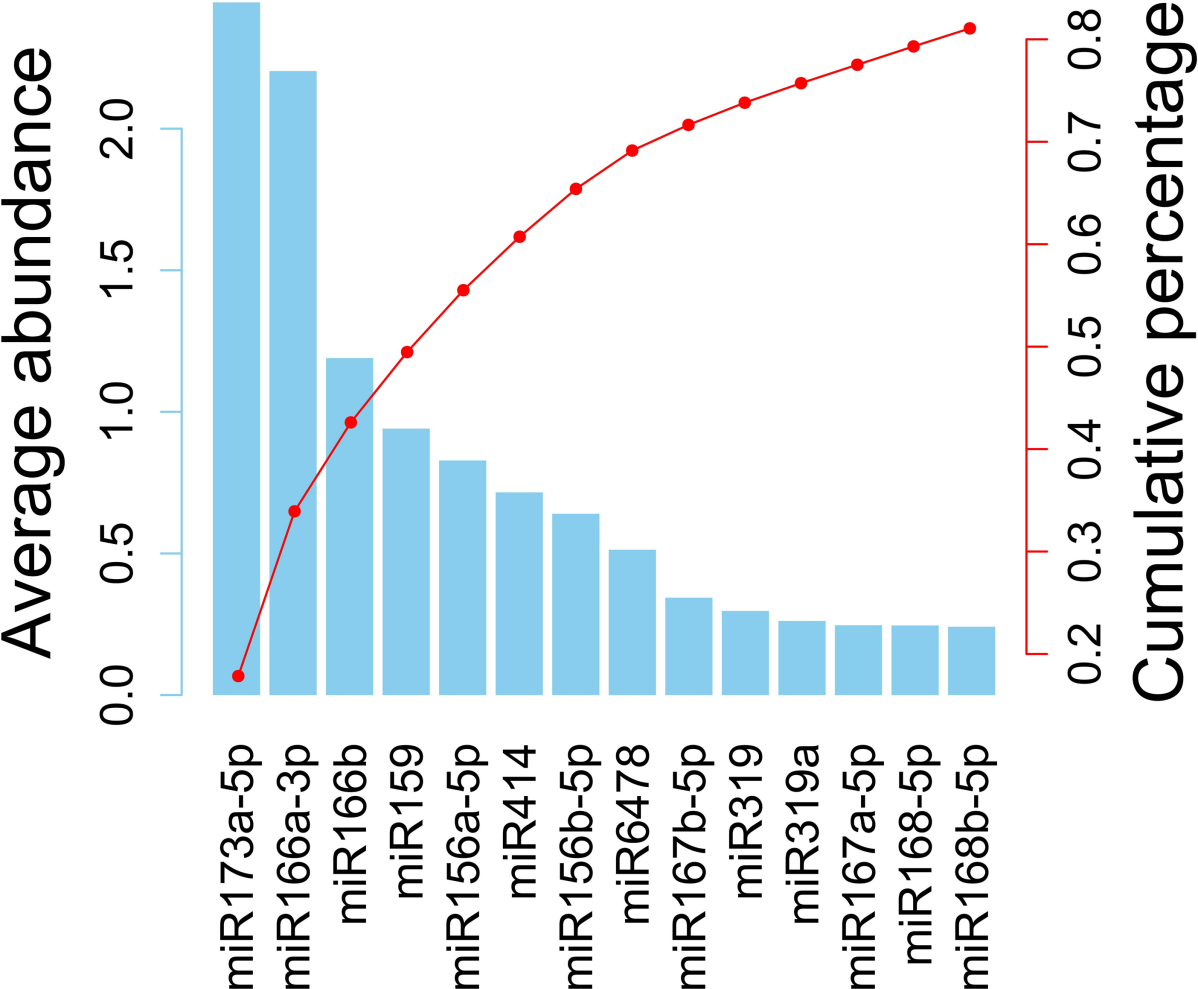
The average abundances and the percentages of the top 14 plant miRNA detected in human samples. The left Y axis denotes the abundance and the right Y axis denotes the cumulative percentage. These miRNAs make up 81.07% of all the plant miRNAs in our samples. Most of plant-derived xenomiRs reported by other papers are included in these 14 types of plant miRNAs, such as miR156, miR159, miR166, miR167 and miR168.

After analysis by our pipeline, a total of 166 types of plant miRNAs were detected in more than half of all the small RNA sequencing data from all 10 types of the body fluids/tissues with the exception of 16 human RBC samples (Table 1). Among the identified 166 types of plant miRNAs (S6 Table), the top 14 most abundant types are shown in Fig 2, which represents 81.07% of all the plant miRNA reads detected in all of our samples. It is worth mentioning that most of the plant-derived xenomiRs reported by other papers are included in these 14 plant miRNAs, such as miR156, miR159, miR166, miR167 and miR168 [11, 16, 22, 24, 68].

### Plant miRNA comparison among yeast, E. coli and human

Sample contamination cannot be avoided entirely in any small RNA sequencing experiment, and for the same reason, it can be regarded as a background distribution in the sequencing data. To date, no plant-derived miRNAs were reported in yeast (*S*. *cerevisiae*) or *E*. *coli*, and hence, it is reasonable to regard the plant miRNA detected in these samples as the background distribution. To characterize this background distribution, we determined plant miRNAs in small RNA sequencing samples of yeast and *E*. *coli* (Tables 2, S7 and S2) using our computational pipeline. In total, 77 plant miRNAs belonging to seven types of plant miRNAs (miR166a-3p, miR166b, miR156a-5p, miR414, miR396b-5p, miR396e, and miR166u) were detected in 10 out of 41 of the yeast and *E*. *coli* samples (Table 2).

**Table 2:** Microbiome sample list.

Species	No. of samples	No. of samples containing plant miRNA(% ^a^)	No. of reads mapped to plant miRNA (Average abundance)
Yeast	28	8 (28.57%)	74 (0.0623)
E. coli	15	3 (20%)	3 (0.0492)
**Sum**	**43**	**11 (25.58%)**	**77 (0.0558)**

^a^ Values in the parentheses were the percentage of samples containing plant miRNAs.

**Fig 3:**
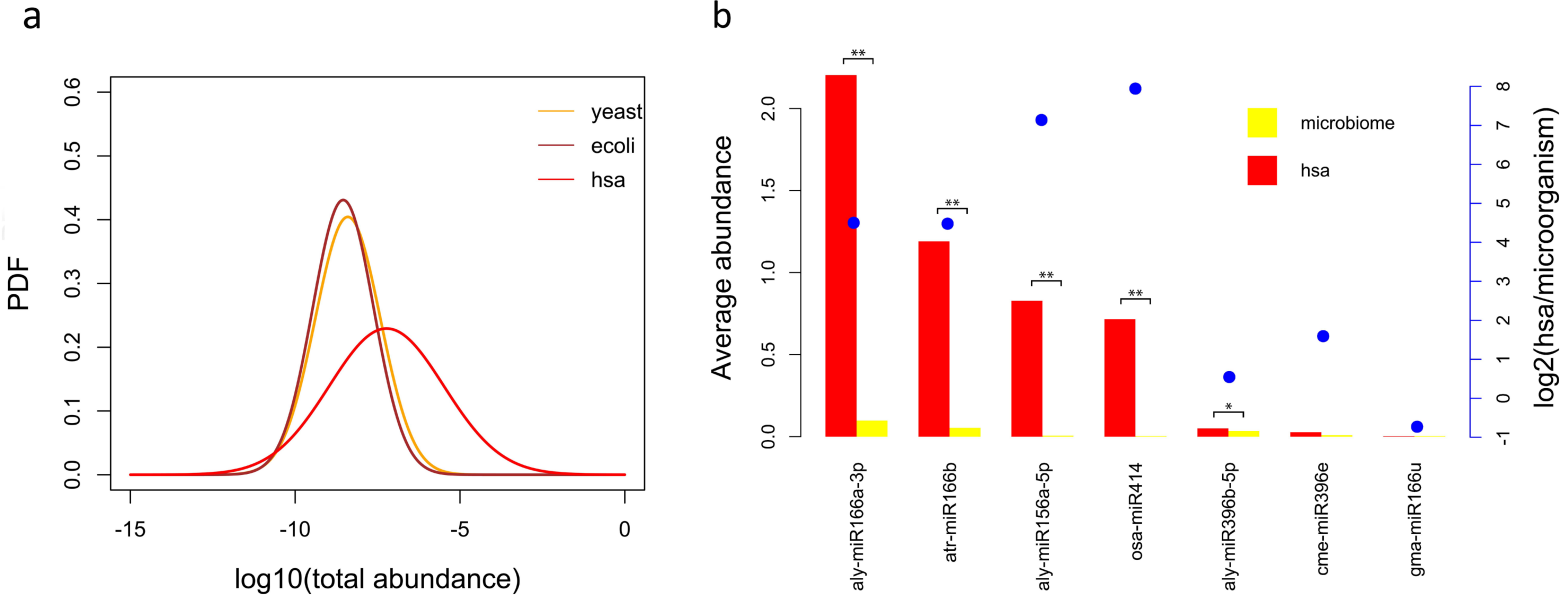
Plant miRNA abundance comparison between human and yeast/E. coli shows significant differences. (a) The logarithm of plant miRNA total abundance in human, yeast and *E*. *coli* were fitted into Gaussian distributions. The abundance distributions of yeast and *E*. *coli* are very similar, but the abundance distribution in humans is obviously different. (b) Abundance comparison of each plant miRNA between human and microbiome (including yeast and *E*. *coli*). The left Y axis denotes abundance and the right Y axis denotes the log2(human average abundance/microbiome average abundance). The abundance differences of miR166a–3p, miR166b, miR156a−5p and miR396b–5p between human and microbiome were significant. The * denotes 0.05 < p <0.1 and ** denotes p < 0.05.

Comparing the plant miRNA abundances between human and yeast/*E*. *coli* samples in Tables 1 and 2, it is evident that the average abundance of plant miRNA in both yeast or *E*. *coli* is far less than that in human. We assume the denary logarithms of plant miRNA abundances of human, yeast and *E*. *coli* samples follow Gaussian distributions as shown in Fig 3A (see “Materials and Methods”). The abundance distributions of yeast and *E*. *coli* are very similar, but they are very different from that in humans (Fig 3A). T-test also showed that the plant-derived miRNA abundances were not significantly different (p = 0.6534) between yeast and *E*. *coli*, but significantly different (p = 1.025e–05) between human and yeast/*E*. *coli*. This suggests that yeast and *E*. *coli* samples do not contain plant-derived miRNAs except for those in plant miRNA background distribution, while human samples do.

We also compared the abundance of each type of plant miRNA detected in yeast/*E*. *coli* between human and yeast/*E*. *coli*, which is shown in Fig 3B. When the abundance of a plant miRNA is relative high, especially when the abundance is higher than 0.05, the difference between human and yeast/*E*. *coli* is significant based on the T-test and usually the abundance of plant miRNA in the human is several times higher than that in the yeast/*E*. *coli*. Therefore, in the following analysis, we only used those plant miRNAs in human samples with average abundances of more than 0.05 unless stated otherwise, which resulted in 24 types of plant miRNAs in total (S3 Table). The abundance difference of each type of plant miRNA between yeast and *E*. *coli* was insignificant (T-test, p > 0.1, S1 Fig).

For further confirming that plant miRNAs in human samples are different from the background distribution, we also used human miRNAs detected in the ath small RNA sequencing data for more comparison. Human miRNAs were screened from 62 ath samples (S3 and S8 Tables) using a similar pipeline described in Materials and Methods, and their abundances along with plant miRNA abundances in human samples and microbiome (yeast/*E*. *coli*) samples were shown in S2 Fig. The human miRNA abundances in the ath samples are significantly lower than the plant miRNA abundances in human samples (T-test, p = 0.00132), but not significantly different from the plant miRNA abundances in microbiome samples (T-test, p = 0.1939). This suggests that plant miRNAs in human samples are significantly above the background noises.

### Plant miRNA profile comparison between human and plant

**Fig 4:**
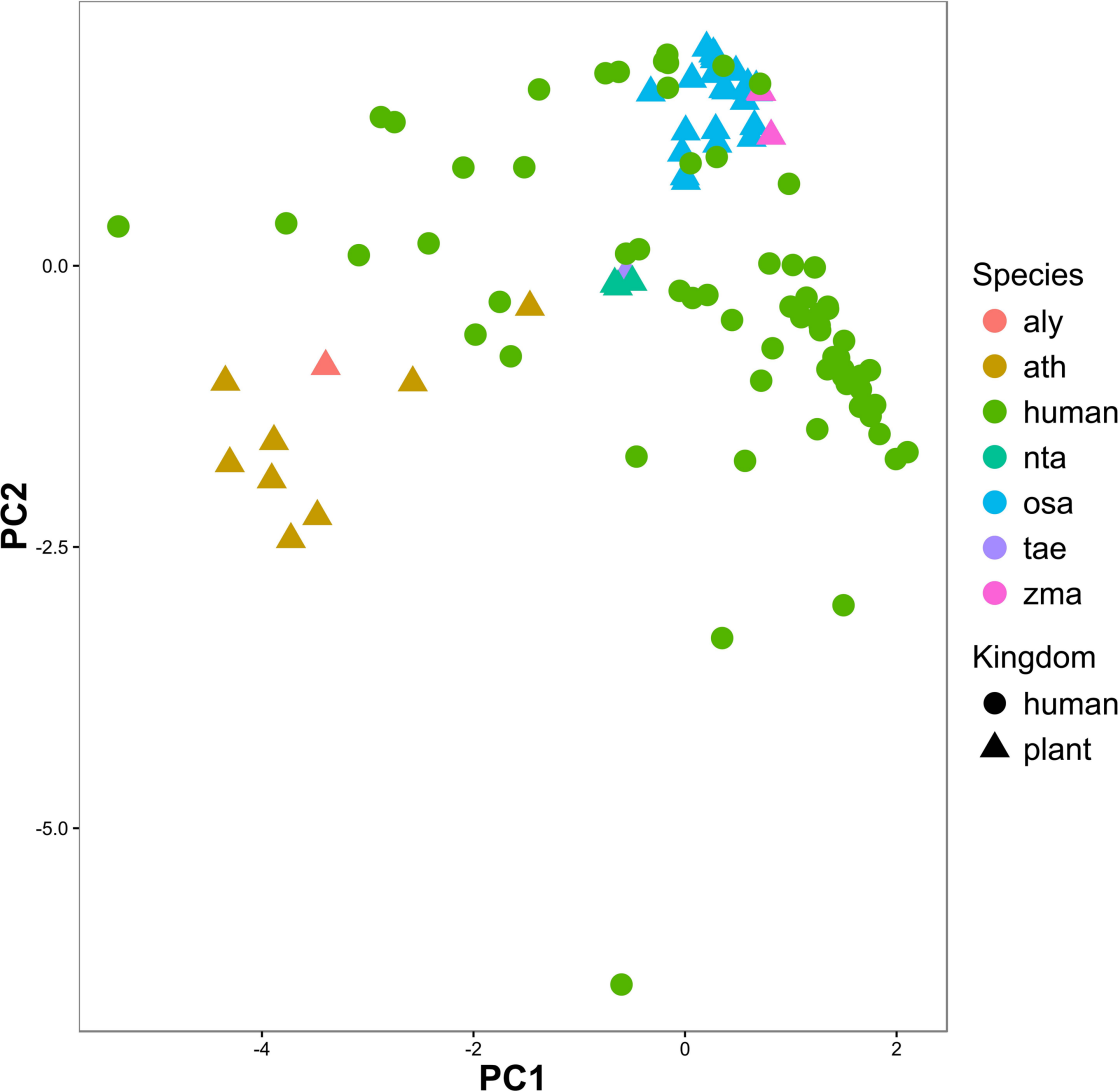
PCA based on common plant miRNAs from humans and six types of plant show that samples from plants could be clustered into three subgroups while most human samples were separated from plant samples, especially ath and aly samples.

We compared the miRNA profiles identified from human samples and miRNA expression profiles from plant samples. Forty-three small RNA sequencing samples were selected from 6 plants (aly, ath, nta, osa, tae and zma) as the representation of plants, and from 37 human samples as the representation of humans (see “Materials and Methods”). PCA was conducted based on these human and plant samples, and the first two components of PCA show that the samples from plants could be roughly clustered into three subgroups while most human samples were separated from the plant samples, especially separated from the ath and aly samples (Fig 4). However, some human samples were clustered tightly with plant samples. Marginal two-sample rank sum test also shows the differences between human samples and plant samples to be significant (p = 6.52e–13). This suggests that the plant miRNAs we obtained from human samples may not result from plant-originated cross-contamination.

### Plant miRNA profile comparison among body fluids/tissues

**Fig 5:**
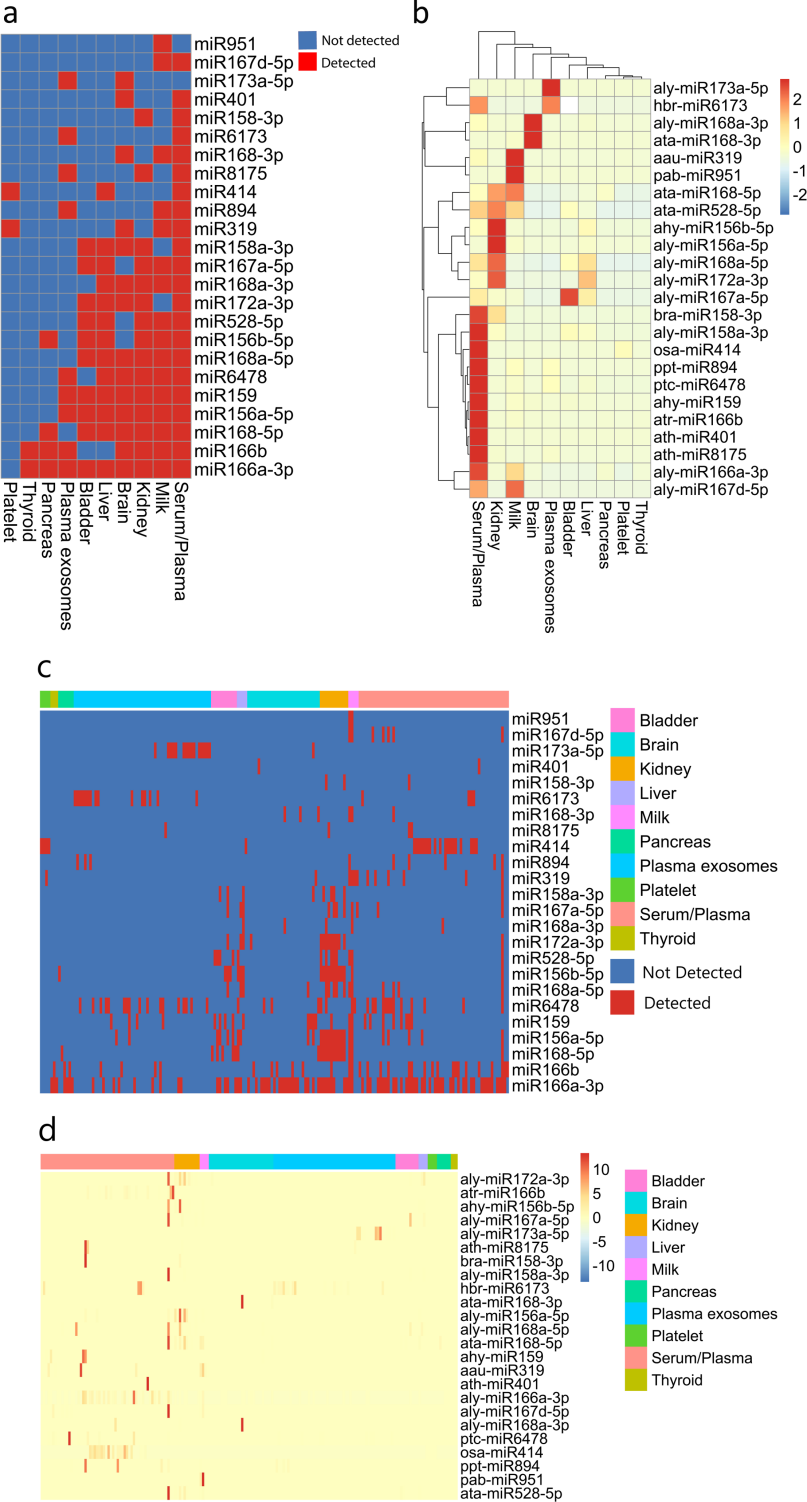
Plant miRNA types and abundances show plant miRNAs in human body are tissue-specific. (a) The plant miRNA types in human body fluids/tissues are different. Serum/plasma samples contained 22 out of 24 most abundant plant miRNAs, while in thyroid and platelet samples, only two types of plant miRNA were detected. Some plant miRNAs covered almost all the body fluids/tissues, such as aly-miR166a–3p, but some miRNAs only appeared in specific body fluids/tissues, for example, pab-miR951 only appeared in milk samples. (b) Hierarchical clustering showed plant miRNA abundances in different human body fluids/tissues had different patterns. The plant miRNA abundance values were scaled by row and Euclidean distance was used as distance measurement between samples. (c) Plant miRNA types in each human sample. The orders of body fluids/tissue and miRNA are identical to those in Fig 5A. (d) Abundance of plant miRNAs in each human sample. The plant miRNA abundance values were scaled by row, and the orders of body fluids/tissues and miRNA are identical to those in Fig 5B.

Many endogenous miRNAs are tissue-specific in both human and plant [69]. We also found that the average abundances of plant miRNAs, especially the five most abundant plant miRNAs, in different body fluids/tissues were also very different (Tables 1 and S9). Hence, plant miRNAs detected in human samples are tissue-specific. The major types of plant miRNAs detected in our body fluids/tissues samples are shown in Fig 5A and 5C. It can be found that serum/plasma samples contained 22 out of 24 of the most abundant plant miRNAs, while in thyroid and platelet samples, only 2 types of plant miRNA were detected. Some of plant miRNAs appeared in almost all the body fluids/tissues, such as aly-miR166a–3p, while some miRNAs only appeared in a few body fluids/tissues, for example, pab–miR951, which only appeared in milk samples. Furthermore, hierarchical clustering showed that the abundance of plant miRNAs had distinguished patterns in different body fluids/tissues (Fig 5B and 5D). Hierarchical clustering based on all 166 types of plant miRNAs detected in all human samples is shown in S3 Fig. To overcome the loss of information, hypothesis tests were performed, and the results show that the differences between plant miRNA profiles identified in human and those in native plants are significant (MANOVA, p = 2.874e–10). However, as we expected, the difference between serum samples and plasma samples was insignificant (MANOVA, p = 0.180). The total abundances of all 166 types of plant miRNAs from different human body fluids/tissues were also significantly different (S4 Fig, ANOVA, p = 0.0156), in which the medium abundance of serum/plasma was the highest and that of brain was the lowest. We also calculated the Pearson correlations (See Materials and Methods) between samples from the same or different body fluids/tissues (S5 Fig). The results showed a stronger correlation between the samples from the same body fluid/tissue than between samples from different body fluids/tissues. T-test also confirmed that the differences were significant (p < 2.2e–16).

We also checked if the presence of plant miRNAs could be explained by the study. We performed hypothesis test on the samples of the same tissue from different studies to test if plant miRNA profiles from different studies were from the same distribution. The results showed that the differences in brain, kidney and pancreas samples from different studies were all insignificant (MANOVA, p>0.05), which suggests that the specificity could not be explained by the study. However, the differences of serum/plasma and exosomes in plasma samples from different studies are significant (MANOVA, p<0.05), which may be due to the different regions of the samples collected by different laboratories.

## Discussion

We carried out our study based on a large number of human small RNA sequencing samples collected from public databases. A computational practical pipeline was built to determine potential plant miRNAs in our data. We analyzed comprehensive plant miRNA profiles using bioinformatics and statistical tools, and yielded reliable evidence to support the plant-derived xenomiR hypothesis.

It is worth noting that, in our pipeline, we removed all the reads possibly belonging to human RNAs with a relaxed criterion before deciding whether they were from plants. It is obvious that most reads in human small RNA sequencing data should come from human cells. And these reads belonging to human are likely to map to many RNAs of other animals or plants by chance due to the small size of reads and the huge amount of small RNA sequencing data sets, which might cause false-positive mapping to RNAs in other species. However, in recent studies [57, 58], these reads were not filtered out before further analysis.

The modified TPM was used for measuring the abundances of miRNAs in sequencing samples collected in our study. In fact, both the common TPM (TPM = #miRNA reads / #raw reads * 1,000,000) and the modified TPM (TPM = #miRNA reads / #clean reads * 1,000,000) were widely used when analyzing miRNA sequencing data. In our study, we analyzed small RNA sequencing data from human, plant and microbiome, which were sequenced using different protocols and by different instruments. This may introduce additional noises into raw sequencing data, which may make analyses difficult. Hence, we selected modified TPM as the uniform measure of the miRNA abundances of all samples in our study. In addition, many sequencing data (sra format files) were uploaded to GEO after removing adapters, such as SRR4295721; so it is difficult to obtain common TPM values of these samples. S6 Fig represents Fig 3A using common TPM, and these two figures are highly similar with little difference, which would not make any difference on the conclusion of this study.

Many studies reported that the plant miRNA detected in human samples were caused by the plant-originated contamination or sequencing errors [12–15]. The distribution of contamination and sequencing errors should show a stable background distribution across all the sequencing data. However, Fig 3A shows that the abundance of plant miRNA distribution in human samples was much higher than the background distribution (yeast or *E*. *coli*). The abundances of most types of plant miRNAs in humans were also much higher than those in the background distribution, but for miR369e and miR166u, the abundance differences were not significant (Fig 3B). For miR166u, its average abundance in microorganism samples was more than that in human samples, which suggests that the low abundance of plant-derived miRNA in microorganism samples cannot be explained by the controlled environment they cultured. Hence, contamination or sequencing errors (probably in the cases of miR369e and miR166u) indeed exist, but it could not explain all plant miRNAs in detected human samples.

When the average abundances of plant miRNAs in human samples were low, especially lower than 0.05, the difference between plant miRNA distribution in human and background became insignificant (Fig 3B). Hence, we used 0.05 as the abundance cutoff and only 24 types of plant miRNAs were used (S4 Table) with an average abundance above this cutoff for further analysis.

If cross-contamination arises during sequencing, the plant miRNA profiles of cross-contaminated human samples should be similar to that of plant-originated contamination. We compared the plant miRNA profiles of human samples to those in six types of plants that are often used in experiments. The result showed that most human sample profiles were far removed from plant miRNA profiles (Fig 4) and the hypothesis test (marginal two-sample rank sum test, p = 6.52e–13) also confirmed that the plant miRNA profiles were significantly different between humans and plants. Hence, plant miRNAs detected in human samples may not result from cross-contamination. It is interesting that the plant miRNA profiles of human were closer to food crops, such as osa, zma, and tae, instead of Arabidopsis (aly or ath), which are used more widely in experiments as model plants (Fig 4). This supports the plant-derived xenomiR hypothesis. However, some human samples were clustered tightly with plant samples, which suggests that the plant-source contamination in these samples may co-exist with xenomiRs.

Using our pipeline, we found that plant miRNAs could be detected in 10 out of 11 types of body fluids/tissues samples; however, no plant miRNA could be detected in 16 RBC samples from three laboratories (Table 1) even though the criteria for screening were relaxed. This indicates that not all the human tissues contain plant miRNAs. Furthermore, some plant miRNAs only appeared in specific body fluids/tissues, and the types and abundances of plant miRNAs contained in different body fluids/tissue were also significantly different (Fig 5). However, only part of plant miRNAs (aly-miR156a-5p, aly-miR166a-3p, aly-miR172a-3p, osa-miR414, pab-miR951, p < 10e-6, MANOVA) in Fig 5 showed strong fluid/tissue specificity, and some of plant miRNAs did not, such as aly-miR168a-3p and ata-miR168-3p, which only presented in 2 and 3 samples, respectively. These results suggest that some plant miRNAs in human body may be fluids/tissues specific; however, it still needs further confirmation by enough number of samples and more rigorous statistics tests to be conclusive. Although we did not detect any plant miRNAs in our RBC samples, this did not mean that the background distribution of plant miRNAs is not present in RBC samples. The absence of plant miRNAs in RBC samples may be caused by an insufficient number of samples (16 RBC small RNA sequencing samples from three studies). Brain cells are separated from the blood by the brain-blood barrier [70], which rigorously controls molecules to enter the center nervous system. A recent study by Lydia et al. [71] reported that exosomes could deliver siRNA to the brain in mice; however, the mechanism remains to be elucidated [72]. In addition, Jing et al. [24] found plant-derived xenomiRs in human umbilical cord blood and amniotic fluid. They proposed xenomiR (including miRNA and siRNA) could transfer through the placenta by microvesicles, which indicates that plant-derived miRNAs can pass through human barriers. Taken together, the encapsulated plant-derived miRNAs in the blood are likely to transfer through human barriers, as detected in the human brain samples of our study (S4 Fig). Nevertheless, the plant-derived miRNA abundance in the brains is the lowest among all human body fluids or tissues.

Naked miRNAs are easy to be degraded in human systemic circulation [73]. According to many studies [74–76], exosomes are one of the possible approaches by which miRNAs are transported between cells. Microarray and deep-sequencing approaches have revealed that small RNAs in exosomes do not mirror cellular populations, which indicates the involvement of selective sorting mechanisms [74, 77–80]. In addition, exosomes possess surface receptors/ligands and have the potential to selectively interact with specific target cells [81]. Jia et al. [29] also recently reported that the copy number of miR166b variated according to tissue types of silkworms fed with synthetic miR166b. Our finding that plant-derived xenomiRs are tissue specific is in accordance with these studies.

It is worth noting that most related studies detected plant miRNAs in various body fluids/tissues of different animal samples, and they reported both positive and negative results. However, the characteristics of non-human animals and the characteristics of human body fluids/tissues were not considered in these studies. In other words, the results obtained from human samples (body fluids/tissues) may not be reproduced using non-human animals (e.g., insect samples [12]). The background distribution is another important factor, which should be considered. The fact that some plant miRNAs detected in animals could not be detected in their diets [12] may be caused by the inherent plant miRNA background distribution which lies in any sample. According to our results, the average abundance of plant miRNA in human body fluids/tissue was only 6.8 (Table 1), so that when the sequencing depth is not enough, such low abundance transcripts may not be detectable by sequencing machines. In addition, the sequencing procedure is biased against plant miRNAs compared with non-modified animal miRNAs because the 2ʹ-O-methyl modification of the 3ʹ-ends of plant miRNAs results in decreased adaptor ligation efficiency [82]. It is, therefore, not surprising that the sequencing reads of plant miRNAs are low in the mixed plant and animal libraries, including human body fluids/tissue samples. However, Dickinson et al. [15] reported that no significant bias was observed in qPCR-based quantification of RNA oligonucleotide with 2’-O-methyl 3’-end spiked into plasma RNA. In addition, a recent research [26] reported that strawberry fruit FvmiR168 could affect properties of dendritic cells at a concentration three orders of a magnitude lower than that required to induce a similar effect by human miRNAs. Diets are another factor for consideration. Although plant miRNA profiles of same body fluids/tissues are more alike than those of different body fluids/tissues (S5 Fig), many correlation coefficients between same body fluids/tissues are rather low, which may be caused by different diets.

Although our results strongly support the plant-derived xenomiR hypothesis, it is important to note that our results do not imply whether the plant miRNAs have any biological functions or if they could affect phenotype in human. It is still far-fetched to assume that genetically modified crops could have any effects on the human body through plant-derived xenomiR. As a statistical analysis, this study cannot serve as the ultimate proof of the plant-derived xenomiR hypothesis. More well-designed experiments and more rigorous analyses are needed to further investigate this hypothesis. For example, this study can be validated or refuted by radioisotope labeling experiments, where the intact radioactive miRNA molecules that are identified in this study may be detected at the molecular level in the predicted tissue types or observed through medical imaging of an animal body.

## Conclusions

Taken together, plant-derived xenomiR is the most likely explanation of the analyses for a large number of public small RNA sequencing data. This study provides the first statistically solid evidence on plant-derived xenomiR profiles using large-scale data to validate the plant-derived xenomiR hypothesis in humans. It gives a first atlas of xenomiR distributions in different tissue types. It also suggests specific miRNAs that can be synthesized with isotopes for *in situ* detection after feeding them to animals, as a more rigorous validation of this hypothesis.

## Supporting information

S1 Fig Plant miRNA abundance comparison between yeast and *E*. *coli*. The abundance differences of all seven types of plant miRNA in our samples were not significant (T-test, p > 0.1) between yeast and *E*. *coli*.
(JPEG)

S2 Fig Abundance comparison among the human miRNA abundance in the ath sample, the plant miRNA abundance in human sample (hsa), and the plant miRNA abundance microbiome sample. The human miRNA abundance in the ath samples is significantly lower than the plant miRNA abundance in human samples (T-test, p < 0.05), but not significantly different from the plant miRNA abundance in microbiome samples (T-test, p > 0.05).
(TIF)

S3 Fig Hierarchical clustering based on all 166 types of plant miRNAs in human body fluids/tissues showed plant miRNA abundance of different body fluids/tissues had different patterns. The plant miRNA abundance values were scaled by row and Euclidean distance was used as distance measurement between samples.
(JPEG)

S4 Fig Total abundance of plant miRNA from different body fluids/tissues showed significant difference (ANOVA, p = 0.0156). The medium abundance of serum/plasma was the highest and that of brain was the lowest.
(JPEG)

S5 Fig Pearson correlation distributions of sample pairs from a) the same body fluid/tissue and b) different body fluids/tissues. The histograms were drawn with 12 bins (from −0.2 to 1) with an interval of 0.1.
(TIF)

S6 Fig Plant miRNA abundance comparison between human and yeast/*E*. *coli* using common TPM. The logarithm of plant miRNA total abundances in human, yeast and *E*. *coli* were fitted into Gaussian distributions. The distributions are highly similar with those in Fig 3A, in which the modified TPM was used as abundance measurement.
(TIF)

S1 Table Human sample list. (XLSX)

S2 Table Microorganism sample list. (XLSX)

S3 Table ath sample list. (XLSX)

S4 Table 24 plant miRNAs with abundance more 0.05. (XLSX)

S5 Table Abundance of each human sample. (XLSX)

S6 Table 166 plant miRNAs detected in human samples. (XLSX)

S7 Table Abundance of each yeast/*E*. *coli* sample. (XLSX)

S8 Table Abundance of each ath sample. (XLSX)

S9 Table Top 5 plant miRNAs in human body fluids/tissues. (XLSX)
