# Correction: Evidence for plant-derived xenomiRs based on a large-scale analysis of public small RNA sequencing data from human samples

**DOI:** 10.1371/journal.pone.0224537

**Published:** 2019-10-24

**Authors:** Qi Zhao, Yuanning Liu, Ning Zhang, Menghan Hu, Hao Zhang, Trupti Joshi, Dong Xu

After publication of this article [1], concerns were raised about the article. Specifically:

1. Counts per million results are not provided for each miRNA and sample. Instead, only aggregate counts of all putative plant miRNAs per sample are reported in the relevant tables such as S5 Table.
2. Single-mismatch mappings were included in the results, instead of rpms for zero-mismatch mapping.
3. The aggregate results of S5 Table cannot be reconciled with Fig 2 as currently presented.
4. A T-test comparison of 1) the abundance of plant miRNA in human samples, as reported in S5 Table and 2) the abundance of human RNA in plant samples, as reported in S8 Table, should give a *p* value of 0.03, not 0.00132 as stated in the results. S8 Table contains what appears to be incorrectly calculated “TPM” values; to use the notation in the article, S8 Table reports P/T, not P/Tx10^6 as in the other tables. Furthermore, the six datasets from GSE84728 appear to be degradome, not small RNA, sequencing, so it seems unclear if they are appropriate for comparison. Excluding them from the analysis, the T-test result should be >0.1.
5. Reference 27 is included in the list of studies that purportedly “identified plant miRNAs in human or animal body fluids/tissues.” Reference 27 was retracted by the authors in May 2017 after it was discovered that the primers used in the study did not work. As described in the retraction, when the study was re-done with correct primers, no miRNA uptake was observed.
6. Concerns were raised regarding the statement that the statistical analyses performed in the studies of Kang et al [57] and Zheng et al [58] were not rigorous enough.
7. The study referenced as [14] did not use “two primate plasma samples” as stated, instead, they examined eight plasma samples: four longitudinal samples from each of two subjects.

The authors provide the IDs of all the raw sequencing data and their processing pipeline in the Materials and Methods section of [1]. This data can be downloaded via the GEO database using the script offered in the article, and the same results will be obtained if the pipeline is carried out.

Additionally, the authors state that they fully considered the number of mismatches when processing their sRNA sequencing samples. The authors state that generally, most reads (>90% usually) in small RNA sequencing data are from endogenous miRNAs/rRNAs/tRNAs, and that according to common standards, one or two mismatches are allowed when mapping miRNA sequences. The authors allowed two mismatches for the above three species of RNAs, which is loose enough to avoid false-positives. Conversely, when identifying exogenous plant-derived miRNA (xenomiR), the reads must 1) be less than one mismatch and 2) satisfy three other criteria (see the Materials and Methods section of [1]), which is stringent enough to make sure that the reads were more likely from plant miRNAs. It is likely that many false-negatives were ruled out from the datasets.

Furthermore, the authors explain that S5 Table shows plant-derived miRNA abundances of all the samples including both positive and negative samples. In Table 1, the authors grouped their samples by species of body fluids/tissues and showed the average abundance of each including both positive and negative samples, as seen in the last column. The authors also showed the percentages of samples containing plant-derived miRNAs in each species in the third column of Table 1.

As indicated, not all samples contained plant-derived miRNAs. For instance, no plant-derived miRNAs were found in the red blood cells. Fig 2 only showed the top 14 most abundant plant-derived miRNAs. They represented more than 80% of all the plant miRNAs in all samples. This figure accounts only for the plant miRNAs discovered in human samples, and hence, the abundance of each miRNA was calculated from only the samples containing plant-derived miRNAs. Therefore, there is no conflict between S5 Table and Fig 2. This was not intended to mislead the readers about plant-derived miRNA abundances, because their detailed abundances were shown in both Table 1 and S5 Table.

The authors provide a revised S5 Table, which includes the RBC plant miRNA reads, below.

Regarding the fourth point, if the method was performed again as described in this article’s [1] Materials and Methods section, the same results will be obtained. In S8 Table, all the values are indeed P/T. The 6 datasets from GSE84728 are degradome; however, even if these six datasets were removed from the analysis, the T-test result is still less than 0.01 (T-test, p = 0.0029), which does not affect the overall conclusions.

Regarding reference 27, as reported in many papers, although the sequencing samples are often contaminated by RNAs/DNAs of scientists themselves when doing experiments, the plant miRNA abundance in human bodies is significantly greater than the human miRNA abundance in *Arabidopsis thaliana* samples. Additionally, the current authors were not informed that reference 27 had been retracted before submitting to *PLOS ONE*. The authors state that other studies have shown solid evidence of the xenomiR hypothesis [2–6].

Contamination is ubiquitous in experiments; for example, pollen suspended in the air may bring plant miRNAs into animal samples. Since contamination can arrive from multiple sources, it is not strange to detect miRNA from multiple clades in a single sequencing data (as indicated in [58]), especially when the criteria of screening xenomiR are not rigorous enough. In addition, many plants from different clades share common or very similar miRNAs sequence due to homology. Thus, contamination is not the only explanation for identification of plant miRNAs belonging to multiple clades in single sequencing data.

Beyond contamination effects, the authors state that their study attempted to test whether these detected non-human miRNAs were really from the original biological samples or merely contamination. The authors state they used a rigorous pipeline to remove the possible contamination in sequencing data, and only the highly expressed xenomiRs were selected in the following analyses. The authors state that the analysis results have shown that those selected cannot be well explained by contamination (Fig 2, Fig 3a, Fig 3b, and Fig 4), and that the results could well satisfy the xenomiR hypothesis (Fig 5). Therefore, the authors think the plant-derived xenomiR is a better explanation of their observations.

Regarding references 57 and 58, both studies used public databases to investigate plant-derived xenomiRs. Reference 57 first comprehensively studied the exogenous miRNAs in 824 public human sequencing data sets. The authors state that it is obvious that most reads in human small RNA sequencing data should come from human cells, but reference 57 mapped many of the reads to other animals or plants and concluded that this was the result of contamination. The study did not exclude the possibility that these reads mapped to many RNAs of other animals or plants by chance due to the short lengths of reads and the huge amount of small RNA sequencing data sets, which might cause false-positive mapping to RNAs in other species. In the pipeline presented in [1], the authors removed all the reads possibly belonging to human RNAs with a relaxed criterion before deciding whether they were from plants.

Reference 58 developed an online exogenous miRNA analysis tool based on a “map and remove” approach, but the detailed parameters were not shown in their paper. However, they reported the average abundance of miR168a was 22,119.9, which the current authors consider to be out of the reasonable range. In addition, the measurement of the xenomiR abundance was calculated using the same method used in [7], which the current authors consider to be appropriate for measuring host miRNA abundance but not for xenomiR. In [1], the authors used modified TPM, making the measurement of xenomiR abundance more accurate.

Finally, the authors agree that the usage of “two primate plasma samples” is inappropriate to describe the samples used in reference 14. However, it does not affect the conclusions of this article [1].

A member of the Editorial Board reviewed the updated table and author responses and confirmed that they support the results and conclusions reported in the published article.

## Supporting information

S5 Table Abundance of each human sample. (XLSX)
