# Correction: Evidence for plant-derived xenomiRs based on a large-scale analysis of public small RNA sequencing data from human samples

**DOI:** 10.1371/journal.pone.0230146

**Published:** 2020-03-11

**Authors:** Qi Zhao, Yuanning Liu, Ning Zhang, Menghan Hu, Hao Zhang, Trupti Joshi, Dong Xu

The references in the Correction notice do not provide direct support to the points made in the text. The authors have supplied updated references. An increasing number of studies have supported the xenomiR hypothesis in recent years. Li et al. [3] reported that continuous administration of a high plant miRNA diet or synthetic miR156 elevated miR156 levels and inhibited the Wnt/β-catenin signalling pathway in mouse intestine, and demonstrated that plant miR156 inhibits intestine cell proliferation by targeting Wnt10b. The same group also reported that miR167e-5p regulated the proliferation of enterocytes *in vitro* [4]. Hou et al. [5] showed that plant miR156a stably presents in healthy human serum. Their *in vitro* studies demonstrated that miR156a directly targeted the junction adhesion molecule-A, and ectopic expression of MiR156a in human aortic endothelial cells reduced inflammatory cytokine-induced monocytes adhesion by suppressing JAM-A. Moreover, in addition to the two studies mentioned in our paper [6; 7], one more study has shown that breast milk contains plant derived microRNAs (miR166a, miR156a, miR157a, miR172a, miR168a) and the concentration of these microRNAs were measured [8].

Not limited to mammals, plant-derived xenomiRs also exist and function in insects and bacteria. Zhu et al. [9] reported plant miRNAs miR162a in bee bread targeted amTOR in bees, which delayed development and decreased body and ovary size in honeybees, thereby preventing larval differentiation into queens and inducing development into worker bees. Another research suggested that exosome-like extracellular vesicles secreted from *Arabidopsis* cells transported small RNAs into the fungal pathogen *B*. *cinerea*, which suppressed its pathogenicity by silencing fungal virulence genes [10].

A number of studies have investigated plant derived xenomiRs using computational approaches. Patel et al. [11] identified conserved and novel *O*. *basilicum* miRNAs from expressed sequenced tags using computational approaches, and predicted their targets and potential functions on human. Using bioinformatics tools and databases, Ergün [12] investigated cross-kingdom gene regulation via miRNAs of *H*. *perforatum* flower dietetically absorbed to define potential biomarkers for prostate cancer. Yu et al. [13] developed a related database MepmiRDB, which collected thousands of potential miRNAs/xenomiRs belonging to 29 medicinal plant species. Zhao et al. [14] analyzed the differences between 166 plant-derived xenomiRs and 942 non-xenomiRs, and trained a 1D-CNN model to predicted other possible xenomiRs from unlabeled plant miRNA sequences.

These studies not only supported the xenomiR hypothesis from different approaches and species, but also started to explore the function, mechanism and medicinal value of plant derived xenomiRs. More related researches were well-reviewed in two recent papers [15, 16].

The authors would also clarify the type of t-test used in the comparison of abundance values of plant miRNAs in human samples (S5 Table) and those of human miRNAs in *Arabidopsis* samples (S8 Table). The authors state that they used “independent samples Student’s t-test” when comparing abundances between the plant miRNA in human samples and human miRNAs in *Arabidopsis* samples. Here, the denary logarithm of plant miRNA abundances of human samples and human miRNAs of *Arabidopsis* samples were assumed to follow Gaussian distributions.

When the abundances of plant derived miRNAs in human samples were analyzed, only the top 24 most abundant plant miRNAs (S3 Table) with abundance more than 0.05 were used, which include most of the plant miRNAs reported by other studies. In many samples, the abundance values of these 24 types of miRNA were 0, and none of these were not excluded. For computational convenience, a very small pseudo-abundance was added to all abundance values (see the Materials and methods section in the original article [1]). The authors confirm the resulting p-values are correct except the p-value that was corrected in [2].
