# Supplementary figures and images for: Evidence for plant-derived xenomiRs based on a large-scale analysis of public small RNA sequencing data from human samples
Source: PLoS One. 2018 Jun 27;13(6):e0187519. doi: 10.1371/journal.pone.0187519 (PMC6021041; doi:10.1371/journal.pone.0187519)

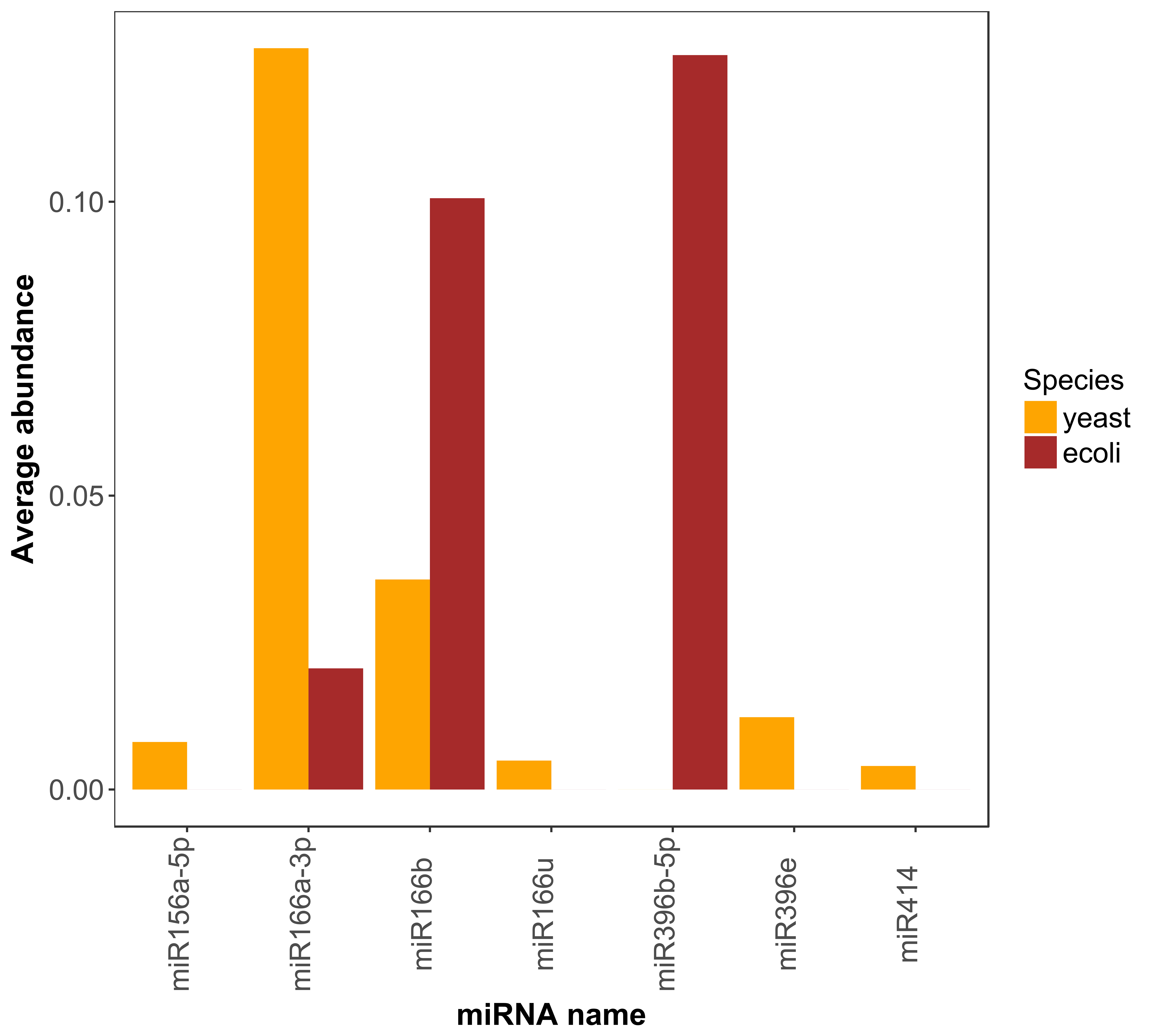

Supplement: S1 Fig — The abundance differences of all seven types of plant miRNA in our samples were not significant (T-test, p > 0.1) between yeast and E. coli. (JPEG) [file pone.0187519.s001.jpeg]

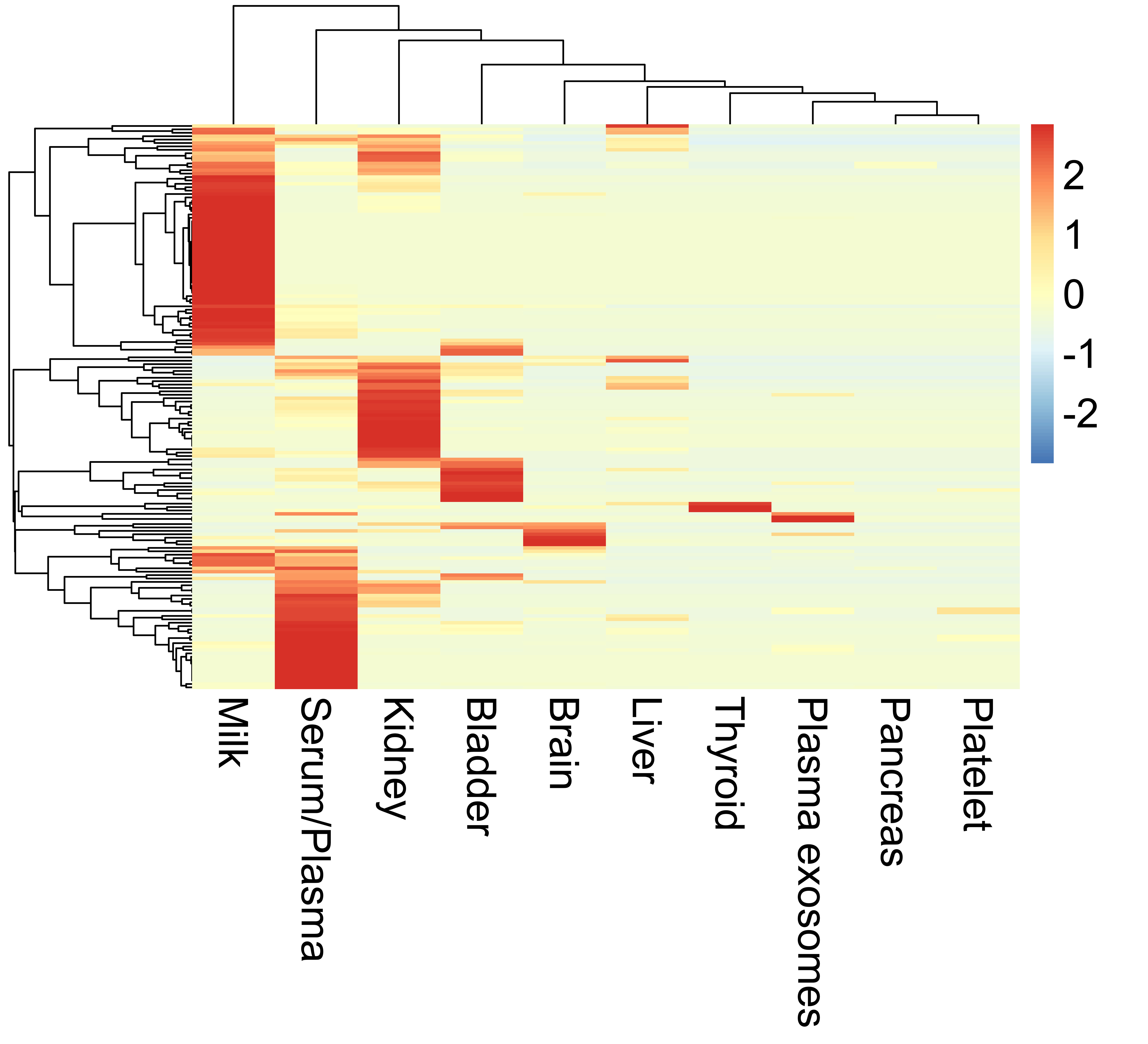

Supplement: S3 Fig — The plant miRNA abundance values were scaled by row and Euclidean distance was used as distance measurement between samples. (JPEG) [file pone.0187519.s003.jpeg]

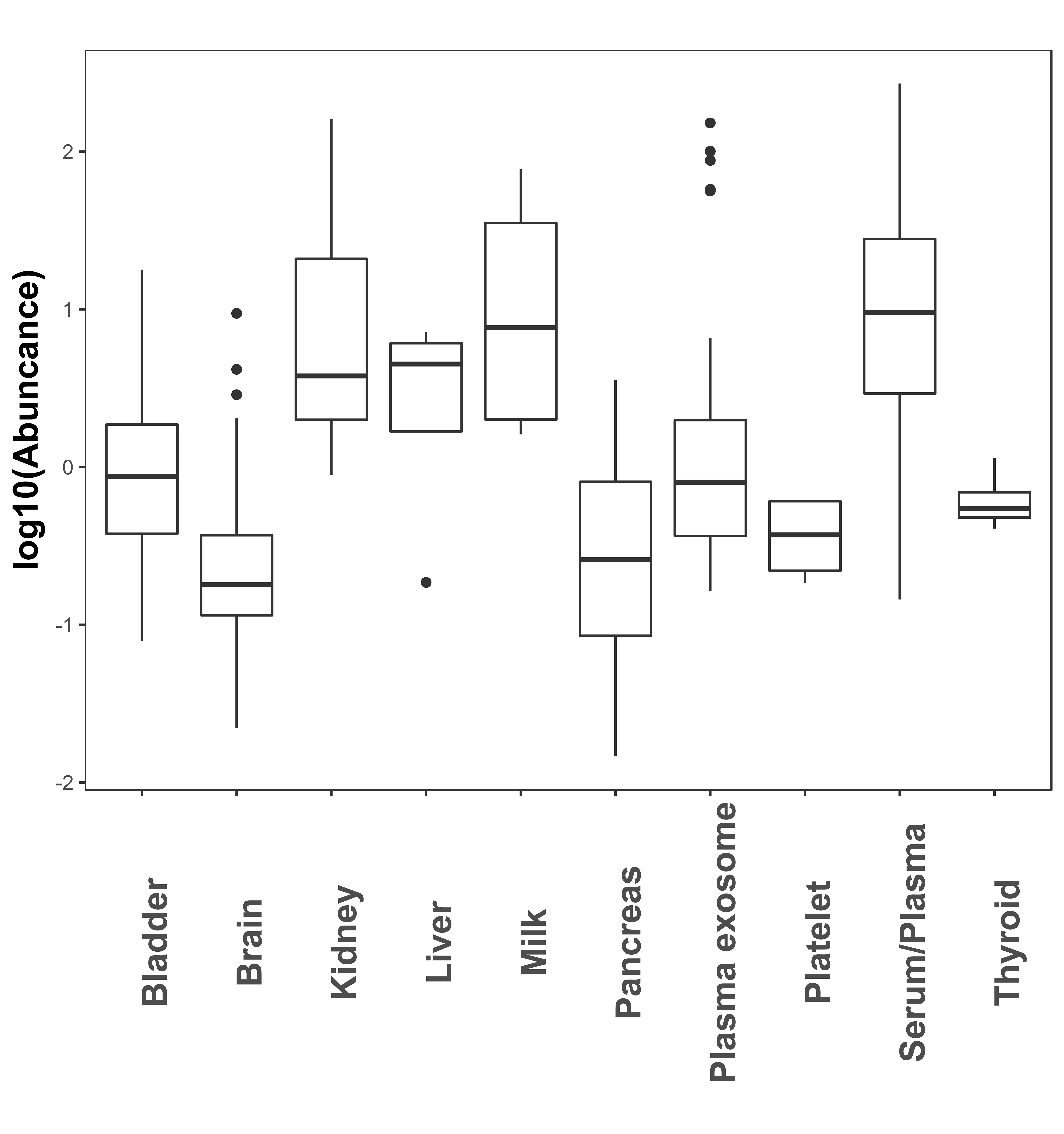

Supplement: S4 Fig — The medium abundance of serum/plasma was the highest and that of brain was the lowest. (JPEG) [file pone.0187519.s004.jpeg]
